# Transvaginal Hybrid-NOTES procedures—do they have a negative impact on pregnancy and delivery?

**DOI:** 10.1007/s00423-021-02105-z

**Published:** 2021-03-31

**Authors:** Panagiotis Thomaidis, Niklas J. Weltermann, Claudia S. Seefeldt, Dana C. Richards, Axel Sauerwald, Markus M. Heiss, Dirk R. Bulian

**Affiliations:** 1 Department of Abdominal, Tumor, Transplant and Vascular Surgery, Cologne-Merheim Medical Center, Witten/Herdecke University, Ostmerheimer Strasse 200, 51109 Cologne, Germany; 2 Department for Obstetrics and Gynecology, Holweide Hospital, Neufelder Strasse 32, 51067 Cologne, Germany; 3 Department of Gynecology and Obstetrics, St. Marien- Hospital, Hospitalstraße 44, 52353 Dueren, Germany

**Keywords:** Transvaginal NOTES, Cholecystectomy, Complication, Pregnancy, Delivery, Posterior colpotomy

## Abstract

**Purpose:**

We conducted a retrospective observational study in order to identify negative effects of NOTES procedures (Natural Orifice Transluminal Endoscopic Surgery) with transvaginal specimen removal on pregnancy and delivery.

**Methods:**

From the total population of 299 patients in our NOTES registry, we tried to contact the 121 patients who were of reproductive age (≤ 45 years) at the time of a transvaginal NOTES procedure. They were interviewed by telephone regarding their desire for children, post NOTES-operation pregnancies, and type of delivery using a structured questionnaire. The collected data was analyzed and compared with current data.

**Results:**

We were able to contact 76 patients (follow-up rate: 62.8%) with a median follow-up of 77 months after surgery (33–129 months). Twenty of 74 participating patients had a desire for children (27.0%). One of them and another's male partner were diagnosed as infertile. Regarding the remaining 18 patients, 14 became pregnant, and three of them became pregnant twice. Considering these 17 pregnancies, there was one miscarriage (5.9%) and one twin birth (5.9%). On average, childbirth occurred 44 months after the NOTES procedure. With regard to the type of delivery, 10 vaginal births (58.8%) and 7 caesarean sections (41.2%) occurred. Thus, the rate of fulfilled desire for children was 77.8%. Compared with the literature, no difference to the normal course could be detected.

**Conclusion:**

There is no sign that the transvaginal approach in Hybrid-NOTES, with removal of the specimen through the vagina, has a negative effect on conception, the course during pregnancy, or the type of delivery.

## Introduction

The acceptance of operations performed through natural orifices (Natural Orifice Transluminal Endoscopic Surgery; NOTES) is rising, and NOTES has been established as an alternative technique to the traditional laparoscopic procedure. Natural orifices like the vagina, anus, mouth, or urethra are used to get to the intraperitoneal cavity by penetrating an intraperitoneal organ. In industrial nations, the laparoscopic cholecystectomy done in 4-trocar-technique is considered the gold standard for treating symptomatic cholecystolithiasis [1]. Nevertheless, in specialized medical centers, procedures like transvaginal Hybrid-NOTES cholecystectomy (TVC) are performed routinely, particularly to avoid abdominal wall incisions for the removal of the target organ [2]. In a prospective randomized trial, TVC showed comparable results to the gold standard in terms of safety but resulted in less pain, increased satisfaction with the aesthetic outcome, and improved postoperative quality of life in the short term [3]. However, for some gynecologists and surgeons the long-term effects concerning infertility are still causing insecurity around TVC [4].

Frequently asked questions at the preoperative consultation are concerned with the lack of information about the occurrence and course of pregnancy after transvaginal surgery, which causes fear and uncertainty [5]. The aim of this study is to evaluate both successful conceptions and parturition, as well as the occurrence of complications during the pregnancies and miscarriages of women with a desire for children after transvaginal NOTES. Therefore, we retrospectively evaluated all our transvaginal NOTES patients who were of childbearing age at the time of the procedure regarding the abovementioned parameters. Our hypothesis is that the transvaginal approach through the posterior vaginal vault does not affect a later conception and delivery.

## Material and methods

### Patients

Since December 2008, records of all patients who underwent a NOTES procedure at our Department of Abdominal, Tumor, Transplant and Vascular Surgery in the Cologne-Merheim Medical Center, a tertiary health care center, were stored in a database. We started with cholecystectomies and since 2012 have added appendectomies, gastric sleeve resections, and operations of the lower intestinal tract including sigma and small bowel resections to our database. Most of our NOTES procedures were performed transvaginally. The Clavien-Dindo classification (CDC) was used to assess the severity of postoperative complications [6].

All patients of childbearing age who had a transvaginal NOTES procedure up until August 2017 were contacted by phone. We set the age of 45 or younger as an inclusion criterion due to the fact that physiological changes marking the onset of perimenopause begin in women's mid-40s and the median age at onset of late perimenopause is 47.5 years [7–10].

After giving informed consent, the patients were interviewed through our questionnaire about their desire to have children and their success at getting pregnant, as well as about the delivery and its type (Table 1). In cases of successful pregnancies, we asked about the appearance of complications during the course of the pregnancy. We created the questionnaire in advance to cover all appropriate parameters.

**Table 1:** Questionnaire

Appearance of pregnancies after transvaginal NOTES procedure
1. Do you have a desire for children after transvaginal NOTES surgery?
2. Did you try to get pregnant unsuccessfully after transvaginal NOTES surgery?
3. Did you get pregnant (planned/unplanned) after transvaginal NOTES surgery?
4. How many pregnancies occurred after transvaginal NOTES surgery?
5. How many successful childbirths did you have after transvaginal NOTES surgery?
6. What kind of delivery was it? (natural or caesarean section?)
7. Did you have a miscarriage? If yes, how often?
8. When was the date of birth/miscarriage?

### Surgical technique

In addition to the age restriction, a posterior colpotomy with removal of the specimen through the vagina was required for study inclusion. In the period of data collection, every patient underwent routine pre- and postoperative gynecological examinations to capture preoperative risks and monitor postoperative complications caused by the transvaginal access. A gynecological infection detected preoperatively was a contraindication to performing a NOTES procedure. After retrieving the specimen, the posterior colpotomy was closed by using a continuous and tight self-dissolving suture (Vicryl® 2–0 or 0). No drainage was inserted through the colpotomy. All NOTES procedures were performed in the lithotomy position.

Transvaginal/transumbilical Hybrid-NOTES cholecystectomies were performed as described previously in the literature [3]. Transvaginal/transumbilical Hybrid-NOTES appendectomies were completed using the technique of Knuth et al. [11]. For colorectal surgery, as well as for ileocolic resection, small bowel resection, adrenalectomy, splenectomy, and sleeve gastrectomy, first steps, including the preparation of the target organ, were performed laparoscopically. In cases of colorectal and small bowel resections, a 13-mm trocar was inserted through the posterior vaginal vault, and the operative steps of closure and separation of the bowel were performed through this access path. Regarding the right hemicolectomy, restoration of bowel continuity was accomplished by stapling anastomosis through this access. In proven or suspicious malignant cases and in all cholecystectomies and appendectomies, the surgical specimen was placed in a specimen bag. To retrieve the specimen, colpotomy was performed after exposure of the posterior vaginal vault under specula and laparoscopic control. In all surgical interventions, except the cholecystectomy, a double ring wound retractor was then inserted via the colpotomy to facilitate the removal of the specimen. For left-sided colon surgery as well as rectal surgery, the anvil of the circular stapler was inserted transvaginally before closure of the posterior vault, creating the ability of a double-stapling anastomosis. The same surgeon (DRB) was involved in all interventions, so a surgeon-side bias could be avoided.

### Outcome parameter

The primary endpoint of the study was the live-birth rate among women who had a desire to have children after transvaginal NOTES. Secondary endpoints were successful conception, miscarriage rate, type of delivery, and the reasons for a caesarean section. The definition of a successful conception was the appearance of a positive pregnancy test, while the definition of miscarriage was an abortion due to any natural reason. We collected patient-related parameters such as length of follow-up, age at surgery, body mass index (BMI), American Society of Anesthesiologists-classification (ASA), and time between surgery and birth. We also compared the patient-related parameters of contacted and unavailable patients to detect systematic differences between these two groups.

### Statistics

The data collected through the questionnaire was prepared in Microsoft Excel Version 14.0.1 (Microsoft Corp., Redmond, WA, USA) and analyzed in SPSS Statistics 26.0 (IBM Corp., Armonk, NY, USA). Data of continuous variables are expressed as minimum, maximum, and median. Binary and categorical variables are reported as counts and percentages. For the comparison of contacted and unavailable patients, the Mann–Whitney *U *test was used for continuous parameters, such as age at operation, BMI, and follow-up, and the chi-squared test was used for categorical parameters, such as ASA. A *p* value of < 0.05 was considered statistically significant.

## Results

**Fig. 1:**
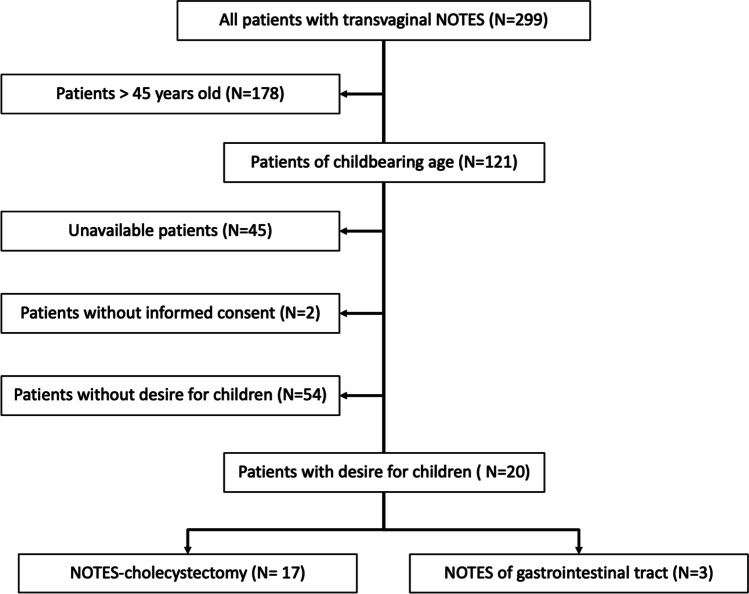
Trial flow diagram

In the period of December 2008 to August 2017, 299 female patients underwent a transvaginal NOTES procedure at our medical center, and 121 of them were of childbearing age (45 years or younger). Specifically, 101 cholecystectomies, 13 appendectomies, 5 bariatric gastric operations, one resection of sigmoid colon due to diverticulitis, and one small bowel operation (resection of Meckel's diverticulum) were performed. According to the database records on these 121 patients, no intraoperative complications or a necessity for a conversion to an alternative technique occurred. Postoperative complications were detected in four (3.3%) patients. Using the Clavien-Dindo classification, these patients developed a grade III complication. Three of them did not require general anesthesia (CDC grade IIIa), and one did (CDC grade IIIb). After a NOTES appendectomy due to a perforated appendicitis, one patient developed an intraabdominal abscess in the right lower abdomen which was treated with antibiotics and a CT-controlled drainage (CDC grade IIIa). After TVC, one case of intraoperative stone loss to the main bile duct was detected and treated by an endoscopic retrograde cholangiopancreaticography (ERCP) with stone extraction postoperatively (CDC grade IIIa). One patient developed a cystic duct insufficiency after TVC, so there was a need for a laparoscopic revision and reclipping of the insufficiency (CDC grade IIIb). The last patient suffered from urinary retention postoperatively and was treated with temporary catheterization (CDC grade IIIa). Forty-five out of 121 (37.2%) women had invalid contact data and were unavailable. The follow-up rate was thereby 62.8%. The median follow-up period of the 76 patients, we were able to contact successfully, was 77 months (33—129 months). Two of 76 (2.6%) declined to take part in our questionnaire. Twenty (27%) of the remaining 74 patients had a desire for children (Fig. 1). All patient characteristics are summarized in Table 2. After comparing the variables of age at operation, BMI, follow-up, and ASA between the group we contacted successfully and the group with invalid contact data, we found no significant differences (*p* = 0.580; *p* = 0.532; *p* = 0.259; *p* = 0.761). One patient was preoperatively diagnosed as infertile of unknown reason. Another patient's male partner was infertile. Out of the 18 remaining patients, 4 (22.2%) had an unfulfilled desire to have children. None of them underwent diagnostics for fertility. One of them already tried unsuccessfully to get pregnant before the operation, while another no longer had the desire for children after trying to get pregnant for a period of 9 months. The third patient had tried to get pregnant for 7 months at the time of the survey interview, while the last one stopped taking birth control for 2 months before the interview. The median age of these four patients was 39.8 years.

**Table 2:** Patient characteristics

	Total patients (*N* = 121)		Contacted (*N* = 76)		Not available (*N* = 45)		Patients with successful conception (*N* = 14)	
	Median	Min./Max.	Median	Min./Max.	Median	Min./Max.	Median	Min./Max.
Age at surgery (years)	35	15/45	34.5	15/45	35	18/45	31.5	22/38
Age at delivery (years)	n.a	n.a	n.a	n.a	n.a	n.a	36	27/41
BMI (kg/m^2^)	27.5	16.1/57.8	28.0	18.3/57.8	26.5	16.1/55.4	28.0	18.5/52.5
ASA	2	1/3	2	1/3	2	1/3	2	1/3
Follow-up (months)	78	33/129	77	33/129	85	36/128	95	40/129

*BMI* body mass index (kg/m^2^); *ASA* Classification of the American Society of Anesthesiologists; *Min*. Minimum; *Max.* Maximum; *n.a.* not applicable

Regarding the remaining 18 patients, fourteen have become pregnant, and three of them have become pregnant twice (Table 3). The rate of pregnancy per patient with a desire for children was 77.8%. The median age of the birthing patients was 36 years. Out of the 17 pregnancies, there was one miscarriage (5.9%). After bariatric NOTES sleeve gastrectomy, one patient gave birth to twins (5.9%) by a transvaginal delivery. With the exception of one patient, the courses of pregnancy were all regular without complications. In this one case, the patient developed gestational diabetes which was conservatively treated with a special diet (patient no. 3).

**Table 3:** Details of patients with childbirths

Patient (type of surgery)	Age at surgery	BMI (kg/m^2^)	ASA	Date of surgery	Date of birth	Length between NOTES and childbirth (months)	Age at childbirth	Type of delivery
01. (TVC)	31	31.1	2	03/2011	06/2013	27	34	Section
02. (TVC)	32	30.8	2	06/2011	02/2014	32	34	Section
03. (TVC)	30	19.6	1	07/2010	06/2016	71	36	Vaginal
04. (TVC)	35	29.3	3	09/2011	03/2015	42	38	Section
05. (TVC)	29	20.8	1	12/2011	06/2015	42	33	Vaginal
06. (TVC)	38	18.5	3	11/2013	10/2015	23	40	Vaginal
07. (NA)	22	23.3	1	11/2012	01/2018	62	27	Section
08. (NSR)	25	45.0	2	04/2013	03/2016; 05/2017	35 49	28 29	Vaginal Section
09. (TVSG)	24	52.5	2	02/2014	01/2017	35	27	Vaginal (twins)
10. (TVC)	37	24.9	2	01/2009	07/2012	42	41	Section
11. (TVC)	33	26.6	2	02/2012	02/2015; 07/2018	36 77	36 39	Vaginal Vaginal
12. (TVC)	35	24.1	1	06/2016	08/2018	26	37	Section
13. (TVC)	33	39.8	2	11/2011	07/2014	32	36	Vaginal
14. (TVC)	30	33.8	2	05/2011	08/2017	75	36	Vaginal

*TVC* transvaginal/transumbilical hybrid NOTES cholecystectomy; *TVSG* transvaginal NOTES sleeve gastrectomy; *NSR* transvaginal NOTES sigma resection; *NA* transvaginal/transumbilical NOTES appendectomy; *BMI* body mass index (kg/m^2^); *ASA* Classification of the American Society of Anesthesiologists

On average, delivery occurred 44 months after the NOTES procedure. In total, there were 10 vaginal births and 7 caesarean sections (Table 3). The rate of performed caesarean sections was 41.2%. The different reasons indicating a caesarean section are summarized in Table 4. No cases were detected where gynecologists recommended a caesarean section due to the previous transvaginal access. The one miscarriage occurred for unknown reasons 23 months after TVC to a 37-year-old patient. The same patient became pregnant after this event and gave birth to a child 19 months after the miscarriage by cesarean section (patient no. 4). Regarding the five patients with postoperative complications, only the patient with postoperative ERCP had a desire for children and became pregnant (patient no. 1). After NOTES sigma resection, one patient gave birth twice (vaginal and cesarean section). Another delivered twice naturally after TVC.

**Table 4:** Indications [17] and reasons of performed caesarean sections

Patient (type of surgery)	Age at surgery	Age at delivery	Reason of performed caesarean section	Indication
01. (TVC)	31	34	Chose the same procedure as in first childbirth	Relative
02. (TVC)	32	34	Section due to patient´s wish	Relative
04. (TVC)	35	38	Section due to inappropriate ratio head size fetus/maternal pelvis	Absolute
07. (NA)	22	27	Section due to high estimated birth weight and shoulder width of the fetus	Absolute
08. (NSR)	25	29	Vaginal try, conversion to section due to obstructed labor	Relative
10. (TVC)	37	41	Section due to surgical removal of one myoma before pregnancy	Relative
12. (TVC)	35	37	Chose the same procedure as in first childbirth	Relative

*TVC* transvaginal/transumbilical hybrid NOTES cholecystectomy; *NA* transvaginal/transumbilical NOTES appendectomy; *NSR* transvaginal NOTES sigma resection

## Discussion

Transvaginal NOTES with extraction of the resected organ through the vagina has shown many advantages over the traditional removal through the abdominal wall. Nevertheless, there are many concerns about getting pregnant, effects during pregnancy, and delivery after posterior colpotomy.

Our analysis includes the first evaluation of transvaginal NOTES procedures with specimen retrieval through the posterior vault of the vagina with regard to its effects on postoperative desire for children, the course of pregnancy, and childbirth. We detected high rates of postoperative pregnancies with a regular course of pregnancy and deliveries without particular complications.

Regarding the latest literature, there is no existing data about fertility and course of pregnancy after transvaginal NOTES. Since 2007, the posterior vault of the vagina has been used as a surgical pathway by an increasing number of general surgeons, e.g., for cholecystectomy, but also for other procedures. Therefore, it is important to collect data on possible complications in addition to the benefits.

On the other hand, data has been published about postoperative sexual function after transvaginal surgery. In a median follow-up of 40 months after TVC, Pohlen et al. did not find any impairment of the female sexual function [12]. Our previous studies support these results with a follow-up of 6 as well as 24 months postoperatively [13, 14]. In a study published in 2013, Tanaka et al. carried out an anonymous questionnaire of female patients with previous transvaginal ovarian cystectomy and found out that there was a high overall satisfaction level and no impairment of fertility [15].

All patients in our study routinely underwent a pre- and postoperative gynecological examination to capture preoperative risks and monitor postoperative complications created by the transvaginal access. However, the analysis of Rossler et al. concludes that preoperative gynecological examination is no longer routinely necessary [16].

In gynecology, using the posterior vault of the vagina is an established method when fertility diagnostics require an intraabdominal view, and it is even used for reconstructive surgery of the internal female genitals [17]. For example, transvaginal chromopertubation is used for diagnosis of fallopian tube patency. Compared with classical fertility diagnostics, in some cases there was a significantly faster occurrence of pregnancy. Liu et al. used the transvaginal approach for tubal reanastomosis and have shown a regained fertility postoperatively [18]. Baekelandt et al. used the transvaginal pathway to detect ectopic pregnancies. In cases of negative exploration of a pregnancy of unknown location, a particular follow-up showed the development of a normal intrauterine pregnancy without adverse effects of the transvaginal approach [19]. It is worth noting that there was no specimen retrieved out of the vagina in all of these procedures, so critics could mention that transvaginal removal could be harmful to the occurrence and course of pregnancy. In our analyses, we have not been able to find evidence for this.

What we have noticed in our results was that only 27% of contacted patients had a desire for children. In our opinion, this small rate is not explained by the demographic change in Germany. Compared with a publication by Sobotka et al., women of childbearing age have a desire for children in 60.1% of all cases [20]. The low rate in our group may be due to the elevated median age of 34.5 years, especially since our group of patients who gave childbirth had a median age of 36 years at the time of delivery. In all our patients, an operative procedure had been performed, and the median age is higher than in Sobotka’s study and therefore not representative. In addition, this low rate in our sample could be explained by women’s distrust of the NOTES procedure. You might suggest, if they had a realistic desire for children, the transvaginal approach in particular may have been refused. Furthermore, Kobiela et al. found that the general attitude of male sexual partners of potential transvaginal NOTES patients towards the procedure was negative, so they would mostly oppose the NOTES technique [21].

Seven out of 17 childbirths were delivered by caesarean section. This rate of 41.2% was higher than the German average rate of caesarean sections which is 32.2% [22]. Comparing the reasons for caesarean sections in our cohort with the reasons published by Mylonas et al., the indications for caesarean sections were relative in most cases (5 out of 7). Even in cases of caesarean sections performed with an absolute indication (2 of 7 in our cohort), there were no connection to the previous colpotomy [23]. In addition, a particular explanation for our higher caesarean section rate could be that women who gave birth had a median age of 36, which is considerably higher than the median of primiparas in Germany (2018: 31.3 years) [24]. The median age of patients with an unfulfilled desire for children was 39.8 years. Concerning this matter, there is no evidence of a higher risk.

In our study a 37-year-old woman had a miscarriage. The age-appropriate miscarriage risk for this patient published by Fretts et al. was 25% [25]. Nineteen months later, the same patient gave an uncomplicated birth, so there was no negative impact of the operation technique. Regarding the patients in our group who became pregnant, the age-appropriate miscarriage risk by Fretts et al. would be 20%, so that the rate of 5.9% in our study is below expected average.

Particularly, due to our retrospective study design and the median follow-up of 78 months, some contact data like phone numbers, stored from the patients at the time of their first hospital stay, were invalid at the time of the survey interview. From our point of view, younger people between 15 and 45 years are associated with a lack of settled lifestyle, so this might provide an adequate explanation. Nevertheless, patient characteristics did not differ significantly between those who were interviewed and those with failed follow-up, so our analysis is representative.

Despite the limitations like the small sample size and the partial loss of follow-up, we think our study has practical implications as well.

## Conclusion

This is the first analysis dealing with the occurrence and course of pregnancies after NOTES procedures with transvaginal specimen retrieval. Regarding our results, we found no negative impact on the occurrence of pregnancy and delivery after undergoing a NOTES procedure through the posterior vaginal vault. From our point of view, the transvaginal approach is also a reliable alternative operation technique for women of childbearing age with a desire for children. To confirm our results, further research including more patients is needed.

## Data Availability

The authors confirm that the data supporting the findings of this study are available within the article.
